# Robotic vs. Laparoscopic Stapling in Robotic Total Mesorectal Excision: A Prospective Cohort Study

**DOI:** 10.7759/cureus.116896

**Published:** 2026-09-25

**Authors:** Rodrigo Moisés de Almeida Leite, Luiz Felipe B Jacomino, Elis Oliveira, Gabrielli De Moura Gonçalves Oliveira, Leonardo Mazie, Francisco Tustumi, Lucas Cata Preta Stozlemburg, Rafael Vaz Pandini, Luiz Eduardo Ceccon Calil de Assumpção, Natalia Bortolini, Alexia Rosa, Luciana Machado, Victor Edmond Seid, Sergio E Araujo

**Affiliations:** 1 Colon and Rectal Surgery, Einstein Hospital Israelita, São Paulo, BRA; 2 Colon and Rectal Surgery, Hospital Israelita Albert Einstein, São Paulo, BRA; 3 Gastrointestinal Surgery, Hospital das Clínicas da Faculdade de Medicina da Universidade de São Paulo (HCFMUSP), São Paulo, BRA; 4 Gastrointestinal Surgery, Universidade de São Paulo, São Paulo, BRA; 5 Colorectal Surgery, Einstein Hospital Israelita, São Paulo, BRA; 6 Robotic Surgery Program, Hospital Israelita Albert Einstein, São Paulo, BRA; 7 Surgical Gastroenterology, Einstein Hospital Israelita, São Paulo, BRA

**Keywords:** colon resection, colorectal cancer, distal rectal cancer, low anterior resection, robotic stapler

## Abstract

Background: Anastomotic leak remains a major complication following total mesorectal excision (TME). While multiple stapler firings during rectal transection are associated with an increased risk of leaks, robotic stapling platforms offer enhanced articulation and precision that may reduce the number of required firings. This study aimed to compare the clinical outcomes and technical efficiency of robotic vs. conventional laparoscopic stapling for rectal transection during robotic TME.

Methods: We conducted a prospective cohort study of 57 adult patients who underwent robotic TME for rectal adenocarcinoma in 2025. We stratified patients by the rectal transection device used: robotic stapling (n = 27) or laparoscopic stapling (n = 30). All patients were enrolled in an ileostomy omission protocol, avoiding routine protective diversion and utilizing postoperative C-reactive protein (CRP) and procalcitonin surveillance to screen for anastomotic leakage. The primary outcome was the proportion of stoma-free patients at 30 days. Secondary outcomes included the number of stapler loads fired, incidence of anastomotic leak, total operative time, and length of in-hospital stay.

Results: Baseline demographic and clinical characteristics were comparable between both cohorts. The 30-day stoma-free rate was significantly higher in the robotic stapling cohort (100%) compared with the laparoscopic cohort (86.6%) (adjusted relative risk (RR): 1.16, 95% confidence interval (CI): 1.03-1.33, p = 0.049). Furthermore, robotic stapling required significantly fewer stapler firings (mean: 1.85 ± 0.45 vs. 2.65 ± 0.67; p < 0.001). The incidence of anastomotic leak was 3.7% in the robotic group vs. 16.6% in the laparoscopic group; however, this trend did not reach statistical significance (RR: 0.22, 95% CI: 0.03-1.78; p = 0.113). Operative time and length of stay did not differ significantly between the groups.

Conclusion: Utilizing a robotic stapler during robotic TME significantly reduces the number of firings required for rectal transection and is associated with a higher success rate of safe ileostomy omission when combined with postoperative biomarker surveillance. Larger randomized trials are warranted to definitively establish whether these technical advantages translate to a statistically significant reduction in anastomotic leak rates.

## Introduction

Total mesorectal excision (TME) is the gold standard surgical approach for rectal cancer. Anastomotic leak remains one of its major postoperative complications, occurring in approximately 5%-20% of patients and adversely affecting short- and long-term outcomes [1,2].

Rectal transection during TME is technically demanding, particularly in the distal rectum, where pelvic narrowness and the limited articulation of conventional laparoscopic linear staplers make it difficult to position the device perpendicular to the bowel axis. This positioning difficulty frequently necessitates multiple stapler firings to complete the transection, and several studies have linked a higher number of firings to an increased risk of anastomotic leak and reported that the number of stapler firings used for rectal division, together with TME itself, was independently associated with anastomotic leak after laparoscopic rectal resection [3]. A subsequent systematic review and meta-analysis confirmed that two firings carry a significantly higher leak risk than a single firing (OR: 2.44, 95% confidence interval (CI): 1.34-4.42) [4]. Staple-line intersections resulting from nonperpendicular rectal transection have also been implicated as potential sites of anastomotic weakness due to compromised distal stump perfusion [5,6]. Concordantly, single-stapled anastomotic techniques, which avoid staple-line crossings altogether, have been associated with a lower anastomotic leak rate than double-stapled techniques after minimally invasive TME for MRI-defined low rectal cancer [7]. Together, these findings provide a mechanistic rationale for simplifying the rectal transection line and minimizing both the number of firings and staple-line intersections.

Beyond TME dissection itself, the robotic platform also enables intracorporeal rectal transection and anastomotic stapling with dedicated robotic instruments (e.g., the SureForm stapler), which combine a 120° cone of wristed articulation with real-time tissue-compression sensing (SmartFire technology) to allow more perpendicular, surgeon-controlled positioning of the device on the distal rectum [8]. These features are proposed to offer greater precision and control during rectal transection and anastomotic creation than conventional laparoscopic staplers, whose more limited articulation can make perpendicular positioning on the distal rectum difficult. However, it remains uncertain whether robotic stapling translates these technical advantages into improved clinical outcomes, specifically anastomotic leak rates and the need for protective ileostomy, compared with conventional laparoscopic stapling.

Historically, a diverting ileostomy has been considered standard practice after low anterior resection with TME, chiefly because it limits the clinical consequences should an anastomotic leak occur. A protective stoma diverts the fecal stream away from the anastomosis, reducing septic contamination and the likelihood of urgent reoperation when a leak develops, and pooled data from randomized trials show that routine diversion lowers the rate of clinically significant leakage [9-11]. These benefits, however, come with a real cost: ileostomy formation carries its own morbidity, including dehydration and acute kidney injury, high-output stoma complications, peristomal skin breakdown, and a measurable psychosocial burden, and it commits the patient to a second operation for stoma reversal, which adds further risk, resource use, and delay to full recovery [9-11].

Weighing these trade-offs, a growing number of studies have moved toward omitting the diverting stoma in selected patients, provided that appropriate risk stratification and structured postoperative surveillance are in place to detect leakage early and intervene before it becomes clinically manifest. This shift from routine to selective diversion motivates the ileostomy-omission protocol adopted in the present study.

This prospective study compares outcomes of patients receiving robotic TME with robotic stapling versus laparoscopic stapling, all included in an ileostomy omission protocol with clinical surveillance based on biomarkers (C-reactive protein (CRP) and procalcitonin), specifically evaluating anastomotic leak rates, stoma necessity, and technical efficiency of stapling.

## Materials and methods

Study oversight

This prospective cohort study used retrospective data collection and was approved by the Institutional Review Board of Einstein Hospital Israelita with a waiver of informed consent (reference numbers: 68495923.6.0000.0071 and 96417925.0.0000.0071) because of the retrospective data abstraction and patient anonymization. In accordance with ethical standards, personal identifiers were anonymized. The study also adhered to the Brazilian General Data Protection Law (Lei Geral de Proteção de Dados) by taking appropriate protective measures. Anonymization techniques were employed to remove any information that could reveal the identities of the subjects.

Cohort abstraction

Adults (18 or more years old) receiving robotic TME for rectal adenocarcinoma were included for analysis. Only robotic-approach cases operated in the year 2025 were included for analysis. All procedures were conducted by the same team of colorectal robotic-certified surgeons from the onco-coloproctology team. Cohorts were divided into two: our historical cohort of laparoscopic rectal stapling (using Echelon 3000 45 mm blue loads, Ethicon, Inc., Cincinnati, OH) by the bedside assistant, or robotic stapling (using SureForm 45 mm green loads, Intuitive Surgical, Inc., Sunnyvale, CA) by the surgeon, after this technology was made available to the surgical team. All patients were included in an ileostomy omission protocol, consisting of no protective diverting loop ileostomy creation and screening for anastomotic leak with third and fifth postoperative doses of CRP and procalcitonin. If the CRP was elevated ≥148 mg/L and/or the procalcitonin was elevated ≥0.18 ng/mL, a CT scan with rectal contrast was performed to exclude anastomotic leak. Upon confirmation of anastomotic leak, the treatment would be individualized by the attending surgeon depending on clinical status and institutional guidelines, including broad-spectrum antibiotics (meropenem), endoscopic negative pressure therapy, and reoperation with terminal colostomy if necessary. Use of a robotic or laparoscopic stapler depended on the availability of the material at the institution. We excluded patients with missing data from the analysis. Unintentionally converted patients, patients with a hand-sewn coloanal anastomosis, or a terminal colostomy without anastomosis were also excluded. Palliative resections were also excluded. The required sample size of 30 patients per group was estimated using Fisher's exact test on the expected difference in proportions of stoma-free patients between study groups.

Inclusion Criteria

Patients diagnosed with rectal adenocarcinoma, robotic TME without unplanned conversion, year of procedure 2025, adult patients (<18 years old), and at least 30 postoperative days of follow-up were included.

Exclusion Criteria

Patients with incomplete medical records, open or laparoscopic approach in TME, and rectal tumors different from adenocarcinoma were excluded.

Covariates

Trained clinical research abstractors collected the covariates of age, gender, preoperative serum albumin, and preoperative serum glycosylated hemoglobin (HBA1c). Tumor characteristics were also collected and abstracted, such as distance from anal verge (in centimeters), MRI clinical staging (T and N), and neoadjuvant radiation therapy.

Primary outcome

Our primary outcome for analysis was a patient being stoma-free within 30 days after surgical intervention.

Secondary outcomes

Secondary analysis included the number of stapler loads fired, incidence of anastomotic leak, total operative time, and length of in-hospital stay. Anastomotic leak was defined as a patient with clinical and radiologic evidence of an anastomotic leak, regardless of treatment modality. Total operative time was defined as the time from the start of robotic docking to the start of anesthesia reversal.

Statistical analysis

We conducted a chi-square test to obtain unadjusted relative risks (RRs) for our primary outcome. We also conducted multivariate Poisson regression to obtain adjusted RRs across groups after adjusting for age, sex, HBA1c, preoperative albumin, clinical T stage, clinical N stage, and neoadjuvant radiation therapy. All analyses were conducted using Stata 18 for Mac, Standard Edition (StataCorp LLC, College Station, TX), license number 401806341276.

## Results

Our cohort included 57 patients: 27 with robotic rectal stapling and 30 with laparoscopic robotic stapling. Median age was 63 years (IQR: 55-72) in the robotic cohort and 62 years (IQR: 53-72) in the laparoscopic stapling cohort. Nutritional status was similar in both cohorts, with a median preoperative albumin of 4.3 mg/dL (IQR: 4-4.6) in the robotic cohort and 4.1 mg/dL in the laparoscopic stapling cohort (IQR: 3.8-4.3). Distance from the anal verge was similar, 8 cm (IQR: 5-10) in the robotic cohort and 10 cm (IQR: 7-14) in the laparoscopic stapling cohort. A minority of patients in both cohorts received neoadjuvant radiation therapy (11.11 % in the robotic and 6.66 % in the laparoscopic cohort). All demographics are summarized in Table 1.

**Table 1:** Demographic characteristics HbA1c: glycosylated hemoglobin; RT: radiation therapy

Variable	Robotic stapling	Laparoscopic stapling	p value
Age, years	63 (55-72)	62 (53-72)	0.567
Male	15 (55.56%)	20 (66.67%)	0.245
Serum albumin	4.3 (4.0-4.6)	4.1 (3.8-4.3)	0.791
HbA1c, %	5.9 (5.6-6.3)	5.9 (5.6-6.3)	0.999
Distance from anal verge, cm	8 (5-10)	10 (7-14)	0.354
Neoadjuvant RT	11.11 %	6.66%	0.553

Primary outcome

Stoma-Free Patients in 30 Days

Most patients remained stoma-free in this study, as all patients in the robotic cohort and 86.6% of patients in the laparoscopic stapling cohort were stoma-free at 30 days. The unadjusted RR for being stoma-free was 1.16 (95% CI 1.03-1.33), p = 0.04. After adjusting for multiple confounders, the RR was 1.15 (95% CI: 1.01-1.33), p = 0.049. Table 2 summarizes the main findings.

**Table 2:** Postoperative outcomes A p value of <0.05 was considered significant SD: standard deviation; CI: confidence interval; RR: relative risk

Outcome	Robotic stapling	Laparoscopic stapling	Relative risks	p value
Operative time, minutes	240 (220-255)	240 (190-300)	-	0.389
Length of stay, days	5 (5-5)	5.5 (4.5-14)	-	0.85
Stapler loads	1.85 (SD: 0.45)	2.65 (SD: 0.67)	-	<0.001
Anastomotic leak	3.7%	16.6%	RR: 0.22 (95% CI: 0.03-1.78)	0.113
Stoma-free patients	100%	86.6%	RR: 1.16 (95% CI: 1.03-1.33)	0.049

Secondary outcomes

Number of Staplers Fired

The mean number of stapler loads fired in the robotic cohort was 1.85 (SD: 0.45), compared with 2.65 (SD: 0.67) in the laparoscopic stapling cohort. There was a significant reduction (p < 0.001) in the mean number of loads fired in the robotic stapling cohort.

Anastomotic Leak

Anastomotic leak had an incidence of 3.7% in the robotic stapling cohort, compared with 16.6% in the laparoscopic stapling cohort (RR: 0.22, 95% CI: 0.27-1.78, p = 0.113).

Operative Time and Length of In-Hospital Stay

The median operative time was 240 minutes (IQR: 220-255) in the robotic stapling cohort and 240 minutes (IQR: 190-300) in the laparoscopic stapling cohort (p = 0.389). The total length of in-hospital stay was five days (IQR: 5-5) in the robotic cohort and 5.5 days (IQR: 4.5-14) in the laparoscopic stapling cohort (p = 0.085).

## Discussion

In this prospective cohort of 57 patients receiving robotic TME, robotic stapling (SureForm 45 mm) was associated with a significant reduction in the number of stapler loads required for rectal transection and a significant increase in the proportion of stoma-free patients at one month, alongside a nonsignificant trend toward fewer anastomotic leaks compared with conventional laparoscopic stapling (Echelon 3000 45 mm).

The main finding was the significant reduction in stapler firings with robotic stapling. This result is consistent with previous comparative studies and may reflect the greater articulation and surgeon-controlled positioning of the robotic device. Tejedor et al. reported that single- or double-firing transection was achieved in a higher proportion of robotic stapler cases than laparoscopic stapler cases (91% vs. 60%, p < 0.001), while anastomotic leak rates did not differ significantly between groups (4% vs. 7%, p > 0.05), closely mirroring the pattern observed in our cohort [8]. A subsequent systematic review and meta-analysis by the same group similarly concluded that robotic staplers reduce the number of firings required for rectal division compared with laparoscopic staplers [12]. More recent quantitative analysis of robotic stapler articulation during rectal transection has demonstrated that surgeons actively use substantial pitch and yaw adjustments to accommodate pelvic anatomy and achieve a more perpendicular, single-plane transection, particularly in technically demanding cases [13]. These findings suggest that articulation is not merely a theoretical device feature but a functional advantage that may improve the consistency and control of distal rectal division. The 120° cone of articulation and real-time tissue-compression sensing (SmartFire) of current-generation robotic staplers plausibly explain this technical advantage, allowing more perpendicular positioning of the device on the distal rectum and reducing the need for multiple applications. Unlike laparoscopic staplers, whose angulation is largely constrained by the assistant positioned at the patient's bedside, robotic firing is controlled directly by the console surgeon, which may allow finer and more deliberate adjustment of stapler angle during transection.

The clinical relevance of reducing stapler firings is supported by observational evidence from laparoscopic rectal surgery. In a multicenter cohort of 1,609 patients, multiple linear stapler firings were identified as an independent risk factor for anastomotic leakage after adjustment for relevant clinical and oncological variables [14]. This association was subsequently reinforced by pooled evidence demonstrating a significantly higher leak risk with two firings compared with a single firing [4]. Nevertheless, these data remain observational and establish association rather than causality [4,14]. A more perpendicular transection line is, in turn, likely to preserve blood supply more evenly across the staple line than an oblique one, in which the acutely angled corners are more prone to relative hypoperfusion; this perfusion-based mechanism, rather than staple-line geometry alone, may plausibly link fewer firings to a lower risk of anastomotic dehiscence, consistent with prior reports associating multiple firings with anastomotic leakage [3,4]. However, this relationship is not universally confirmed: a recent large retrospective series found no correlation between the number of robotic stapler firings and anastomotic leak rate [13], suggesting that the firing-leak relationship established for laparoscopic staplers may not translate identically to the robotic platform, and that other technical factors, such as perfusion, tension-free mobilization, and distal margin, may modulate this association. Our nonsignificant trend toward fewer leaks in the robotic group should therefore be interpreted with caution given the wide confidence interval and small sample size, rather than as definitive evidence of superiority. Looking forward, the ability to control both dissection and stapling from the console also points toward the feasibility of an increasingly "full robotic" TME workflow, in which key technical steps depend less on bedside maneuvers.

The high stoma-free rate observed in both groups supports the feasibility of an ileostomy-omission strategy within a robotic TME program, provided it is paired with structured biomarker surveillance. Only one anastomotic leak occurred in the robotic stapling group, successfully managed with endoluminal vacuum therapy without the need for a diverting stoma, while 86.6% of patients in the laparoscopic stapling group remained stoma-free at one month. Routine omission cannot be advocated, as randomized trials and pooled analyses have demonstrated that diverting stomas reduce clinically significant anastomotic leak and reoperation after low anterior resection. However, emerging prospective evidence supports a risk-adapted policy in carefully selected patients. In the TAilored SToma policY after TME for rectal cancer study, patients classified as low risk using the Anastomotic Failure Observed Risk Score [15] underwent primary anastomosis without diversion, suggesting that selective omission may be feasible when based on formal risk stratification and structured postoperative assessment [16,17]. Within robotic TME specifically, Duhoky et al., using the Key enhancement of the Anastomosis for No-Stoma Surgery technique (staple-line reinforcement and perfusion assessment), demonstrated that a more selective approach to diverting stomas did not increase leak or complication rates [14]. Similarly, Waqas et al. described a robotic anastomotic reinforcement technique specifically developed to avoid a diverting stoma, reporting favorable short-term outcomes [16]. This same question is now being addressed prospectively by the SELective defunctioning Stoma Approach in low anterior resection for rectal cancer trial, a nested randomized controlled study specifically designed to test a selective defunctioning stoma approach and stoma-free survival after TME [17]. These findings, as do our own, suggest that the traditional trade-off described in pooled randomized data, where routine diversion reduces leak rates at the cost of stoma-related morbidity [9,11], can be partially reframed when structured perfusion assessment and close biochemical surveillance to screen for anastomotic leak before clinical deterioration are used to select which patients truly require diversion, rather than applying it routinely to all patients. Because it leads to early intervention, it could also increase the success of nonoperative interventions, such as vacuum endoscopic therapy.

Taken together, the higher proportion of single- or double-firing transections, the more perpendicular and surgeon-controlled positioning afforded by the robotic stapler, and the very low anastomotic leak and stoma rates observed in the robotic stapling group support a broader argument: a fully robotic TME workflow, encompassing both dissection and anastomotic stapling from the console, may produce a more consistently reliable colorectal anastomosis than a hybrid approach that pairs robotic dissection with a conventional laparoscopic stapler. If this greater reliability is confirmed in larger series, it would provide a rational basis for extending ileostomy omission more broadly within full robotic TME, reserving protective diversion for anastomoses judged higher risk on clinical, perfusion, or oncological grounds rather than applying it as a routine safeguard against variability in the stapling step itself.

The safety of the ileostomy-omission protocol in this cohort is plausibly attributable to the systematic postoperative surveillance with CRP and procalcitonin, both of which have well-documented negative predictive value for anastomotic leak in the early postoperative period [18,19]. This surveillance strategy likely allowed early identification of the single leak in the robotic group, enabling timely endoluminal vacuum therapy before clinical deterioration, an approach with a reported pooled success rate of approximately 85% for anastomotic salvage without recourse to a stoma [20]. This outcome illustrates a broader principle increasingly supported in the literature: that omission of a diverting stoma does not require abandoning proactive leak management, but rather substituting mechanical diversion with active biochemical and clinical monitoring paired with early, minimally invasive rescue when needed.

Our findings are broadly consistent with meta-analyses of randomized trials showing no significant difference in anastomotic leak between robotic and laparoscopic TME overall [21], as well as with more recent multicentre data suggesting a possible advantage of the robotic platform specifically related to the quality of rectal stapling rather than to the TME dissection itself [22]. Taken together with the Robotic vs. Laparoscopic Surgery for Middle and Low Rectal Cancer trial's oncological findings [23], our results add a mechanistic layer to this discussion: the potential benefits of robotic TME may be influenced, at least in part, by the stapling device used. By comparing robotic and laparoscopic staplers within an otherwise robotic procedure, our study sought to isolate the stapling strategy as a specific technical variable.

Beyond its potential clinical implications, the reduction in stapler firings may also have direct economic relevance. Holzmacher et al. reported fewer stapler firings per patient with robotic than with laparoscopic stapling (1.86 vs. 2.69, p = 0.001) and, despite the higher unit price of robotic cartridges, lower total stapling expenditure per patient ($473.28 vs. $631.45, p = 0.001). These findings suggest that reduced cartridge use may partially, or under specific institutional pricing structures, completely offset the higher per-load cost of robotic stapling. In our cohort, robotic stapling required approximately 0.8 fewer loads per patient, indicating a plausible mechanism for direct procedural savings. However, we did not collect formal cost data, and Holzmacher et al.'s results come from a single-center retrospective mixed colorectal cohort using a previous-generation robotic stapler; therefore, their cost estimates may not generalize directly to rectal transection with SureForm or to current institutional pricing agreements [24-27].

This study has several limitations. First, the relatively small sample size and the low number of anastomotic leak events limited statistical power and resulted in imprecise estimates for clinical outcomes. Second, allocation to robotic or laparoscopic stapling was not randomized. Third, the single-center design, with all procedures performed by the same experienced robotic colorectal team, may limit the generalizability of the findings to other institutions and stages of the robotic learning curve. Fourth, the 30-day stoma-free endpoint provides only short-term information and does not capture delayed anastomotic complications, anastomotic strictures, subsequent stoma creation, or permanent stoma rates. Finally, no formal cost analysis was performed. These limitations require the clinical findings, particularly those related to anastomotic leakage and stoma avoidance, to be interpreted as hypothesis-generating rather than definitive.

In this prospective cohort, robotic stapling significantly reduced the number of firings required for rectal transection during robotic TME. Although lower rates of anastomotic leakage and stoma creation were observed, these clinical findings remain inconclusive because of the small sample size, limited event count, and nonrandomized design. Larger comparative studies are required to determine whether the technical advantage of robotic stapling translates into clinically meaningful benefit.

## Conclusions

In this prospective cohort study, the use of a robotic stapler in robotic TME was associated with a significant reduction in stapler loads and higher success in ileostomy omission. In addition, the robotic stapler was associated with a nonsignificant trend toward a reduction in anastomotic leak. Small sample size and low incidence of anastomotic leak in this cohort may limit the generalizability of the findings. Further larger trials are required to assess the true value of robotic staplers in robotic TME.

## References

[1] Penna M, Hompes R, Arnold S (2019). Incidence and risk factors for anastomotic failure in 1594 patients treated by transanal total mesorectal excision: results from the international TaTME registry. Ann Surg.

[2] Kapturkiewicz B, Kazanowski M, Lesiak P, Ramsey D, Bebenek M (2026). Incidence and risk factors for anastomotic leakage after transanal total mesorectal excision in a retrospective cohort of 212 patients. Sci Rep.

[3] Ito M, Sugito M, Kobayashi A, Nishizawa Y, Tsunoda Y, Saito N (2008). Relationship between multiple numbers of stapler firings during rectal division and anastomotic leakage after laparoscopic rectal resection. Int J Colorectal Dis.

[4] Balciscueta Z, Uribe N, Caubet L, López M, Torrijo I, Tabet J, Martín MC (2020). Impact of the number of stapler firings on anastomotic leakage in laparoscopic rectal surgery: a systematic review and meta-analysis. Tech Coloproctol.

[5] Roumen RM, Rahusen FT, Wijnen MH, Croiset van Uchelen FA (2000). "Dog ear" formation after double-stapled low anterior resection as a risk factor for anastomotic disruption. Dis Colon Rectum.

[6] Yang Y, Ding F, Xu T, Pan Z, Zhuang J, Liu X, Guan G (2022). Double-stapled anastomosis without "dog-ears" reduces the anastomotic leakage in laparoscopic anterior resection of rectal cancer: a prospective, randomized, controlled study. Front Surg.

[7] Foppa C, Carvello M, Maroli A, Sacchi M, Gramellini M, Montorsi M, Spinelli A (2023). Single-stapled anastomosis is associated with a lower anastomotic leak rate than double-stapled technique after minimally invasive total mesorectal excision for MRI-defined low rectal cancer. Surgery.

[8] Tejedor P, Sagias F, Nock D, Flashman K, Naqvi S, Kandala NL, Khan JS (2020). Advantages of using a robotic stapler in rectal cancer surgery. J Robot Surg.

[9] Pisarska M, Gajewska N, Małczak P (2018). Defunctioning ileostomy reduces leakage rate in rectal cancer surgery - systematic review and meta-analysis. Oncotarget.

[10] Coco C, Tondolo V, Amodio LE, Pafundi DP, Marzi F, Rizzo G (2023). Role and morbidity of protective ileostomy after anterior resection for rectal cancer: one centre experience and review of literature. J Clin Med.

[11] You YN, Hardiman KM, Bafford A (2020). The American Society of Colon and Rectal Surgeons clinical practice guidelines for the management of rectal cancer. Dis Colon Rectum.

[12] Tejedor P, Sagias F, Flashman K, Kandala NL, Khan J (2020). The use of robotic or laparoscopic stapler in rectal cancer surgery: a systematic review and meta-analysis. J Robot Surg.

[13] Cardelli S, Stocchi L, Merchea A (2025). Multiple robotic stapler firings to transect the rectum are not associated with anastomotic leakage. Colorectal Dis.

[14] Duhoky R, Piozzi GN, Rutgers ML, Mykoniatis I, Siddiqi N, Naqvi S, Khan JS (2024). An institutional shift from routine to selective diversion of low anastomosis in robotic TME surgery for rectal cancer patients using the KHANS technique: a single-centre cohort study. J Pers Med.

[15] McKenna NP, Bews KA, Cima RR, Crowson CS, Habermann EB (2020). Development of a risk score to predict anastomotic leak after left-sided colectomy: which patients warrant diversion?. J Gastrointest Surg.

[16] Waqas A, Mykoniatis I, Sidiqi N (2023). Early experience of undertaking robotic assisted total mesorectal excision in rectal resections, avoiding a diverting stoma: Key eHancement of the Anastomosis for No Stoma technique - a case series. Surg Innov.

[17] Rutegård M, Lindsköld M, Jörgren F (2025). SELective defunctioning Stoma Approach in low anterior resection for rectal cancer (SELSA): protocol for a prospective study with a nested randomized clinical trial investigating stoma-free survival without major LARS following total mesorectal excision. Colorectal Dis.

[18] Rama NJ, Lages MC, Guarino MP (2022). Usefulness of serum C-reactive protein and calprotectin for the early detection of colorectal anastomotic leakage: a prospective observational study. World J Gastroenterol.

[19] Giaccaglia V, Salvi PF, Antonelli MS (2016). Procalcitonin reveals early dehiscence in colorectal surgery: the PREDICS study. Ann Surg.

[20] Shalaby M, Emile S, Elfeki H, Sakr A, Wexner SD, Sileri P (2019). Systematic review of endoluminal vacuum-assisted therapy as salvage treatment for rectal anastomotic leakage. BJS Open.

[21] Eltair M, Hajibandeh S, Hajibandeh S (2020). Meta-analysis and trial sequential analysis of robotic versus laparoscopic total mesorectal excision in management of rectal cancer. Int J Colorectal Dis.

[22] de'Angelis N, Marchegiani F, Martínez-Pérez A (2024). Robotic, transanal, and laparoscopic total mesorectal excision for locally advanced mid/low rectal cancer: European multicentre, propensity score-matched study. BJS Open.

[23] Feng Q, Yuan W, Li T (2025). Robotic vs laparoscopic surgery for middle and low rectal cancer: the REAL randomized clinical trial. JAMA.

[24] Park JS, Choi GS, Kim SH (2013). Multicenter analysis of risk factors for anastomotic leakage after laparoscopic rectal cancer excision. Ann Surg.

[25] Emile SH, Khan SM, Garoufalia Z (2022). When is a diverting stoma indicated after low anterior resection? A meta-analysis of randomized trials and meta-regression of the risk factors of leakage and complications in non-diverted patients. J Gastrointest Surg.

[26] Foppa C, Maroli A, Carvello M (2025). Long-term functional outcomes after transanal transection and single-stapled (TTSS) anastomosis for rectal cancer measured by electronic patients reported outcome measures (ePROMs). Eur J Surg Oncol.

[27] Holzmacher JL, Luka S, Aziz M, Amdur RL, Agarwal S, Obias V (2017). The use of robotic and laparoscopic surgical stapling devices during minimally invasive colon and rectal surgery: a comparison. J Laparoendosc Adv Surg Tech.

